# Efficacy of Telitacicept in treating IgA vasculitis nephritis: a two-case report

**DOI:** 10.3389/fimmu.2025.1564242

**Published:** 2025-05-01

**Authors:** Shuang-xi Li, Rui-kang Hu, De-liang Kong, Jing Xu, Qi Bian, Zhi-Yong Guo, Xiao-bin Mei

**Affiliations:** ^1^ Department of Nephrology, Changhai Hospital, The Naval Medical University, Shanghai, China; ^2^ Medical School of Chinese People’s Liberation Army (PLA), Beijing, China

**Keywords:** Telitacicept, IgA vasculitis, APRIL, BAFF, Henoch-Schönlein purpura (HSP)

## Abstract

Telitacicept, a B lymphocyte stimulator/A proliferation-inducing ligand dual-target fusion protein, mainly used for IgA nephropathy and systemic lupus erythematosus. We present two cases where adult patients with IgA vasculitis (IgAV) nephritis were successfully treated with Telitacicept, experienced no adverse reactions during the follow-up. Therefore, Telitacicept represents a promising additional treatment option for patients suffering from IgA vasculitis (IgAV).

## Introduction

IgA vasculitis (IgAV), previously known as Henoch-Schönlein purpura (HSP), is a small vessel vasculitis marked by the deposition of IgA1-dominant immune complexes in the walls of affected blood vessels. IgAV nephritis frequently results in chronic kidney disease and negatively impacts long-term prognosis (1). In adults, kidney involvement is often more severe than in children, and males are affected more than females (2).

The European initiative SHARE recommends oral prednisolone as the first-line treatment for patients with moderate IgAV nephritis (3). Second-line options include mycophenolate mofetil (MMF), cyclophosphamide (CYC), azathioprine (AZA), and cyclosporine for moderate-to-severe cases. However, the therapeutic recommendations are largely based on expert opinion. Identifying suitable drug targets presents enormous challenges for this condition as absent high-level controlled therapeutic trials for IgAV nephritis. Recently studies have reported the high efficacy and safety of Telitacicept in treating IgA nephropathy(IgAN) (4–7). IgAV nephritis is similar to IgA nephritis. Given that Titacercept is safer than traditional drugs, it is worth exploring whether it can be used to treat IgAV nephritis. This study aims to report and analyze two cases of IgAV nephritis that were effectively treated with Telitacicept at our hospital, to explore potential treatment methods for IgAV nephritis further.

## Case report

Patine 1: In 2019, a 32-year-old Chinese male patient developed purpura on his extremities, chest, and back without any clear cause. He went to a local hospital, where laboratory tests showed urine protein levels of 4259.2 mg/24 hours, hematuria at 160 per high power field, albumin at 27 g/L, and serum creatinine at 100 umol/L. He experienced moderate edema in both lower extremities, and his blood pressure (BP) was recorded at 210/120 mmHg. Instead of undergoing a kidney biopsy, he was treated with prednisone at a dose of 40 mg per day (0.6 mg/kg/d) and tacrolimus (TAC) at 2 mg twice daily. He was prescribed an angiotensin II receptor blocker (ARB) to keep his blood pressure below 140/90 mmHg. After intensive treatment, his protein levels in urine were negative. Unfortunately, his disease frequently recurred as he tapered off prednisone and TAC. According to patients, the primary manifestations of each recurrence were skin purpura, heightened proteinuria, increased hematuria, and elevated serum creatinine levels. Consequently, the treatment regimen was changed to include prednisone combined with mycophenolate mofetil (MMF) at a dosage of 0.5 mg twice daily. Nevertheless, his urinary protein excretion (UPE) remained above 1 g/24 hours, and his estimated glomerular filtration rate (eGFR) continued to decrease. He continued taking prednisone for nearly five years, maintaining a daily dosage of 10mg.

He was admitted to our hospital on June 4, 2024, presenting with the following laboratory results: UPE of 3156 mg/24h, urinary protein to creatinine ratio (PCR) of 3112.7 mg/g, urinary albumin to creatinine ratio (ACR) of 2665 mg/g, albumin level of 36 g/L, creatinine level of 156 µmol/L, and eGFR of 47.8 ml/min.

A renal biopsy was conducted, revealing a total of 25 glomeruli. Among these, diffuse mesangial hyperplasia was present, with glomerular sclerosis noted in 12 glomeruli. Additionally, there were 4 instances of segmental sclerosis and 1 crescent. The renal tubules exhibited a 20% degree of atrophy, while the renal interstitium displayed a 20% level of fibrous hyperplasia. Hyaline degeneration of arterioles was also seen. Immunofluorescence showed that IgA was deposited in the mesangial area. Electron density deposition in the mesangial region was observed by immunoelectron microscopy. The final diagnosis from the renal biopsy is type IIIb Henoch-Schönlein nephritis, accompanied by hypertensive renal damage.

To better control the disease, he was treated with prednisone (7.5 mg/d), MMF (0.5 mg every 12 hours), and a subcutaneous injection of Telitacicept at a dosage of 160 mg per week. After 2 months of treatment with Telitacicept, the renal function was stable and UPE decreased of 66.5% (from 3156 to 1895 mg/24h) (**Table 1**). Last follow-up (December 26, 2024), the drug has been used for more than 6 months, the prednisone was tapered to 5mg every other day. The laboratory analyses revealed UPE had decreased to 1273mg/24h, PCR 678mg/g, ACR 592mg/g, albumin 47g/L, creatinine 145umol/L, eGFR 51.9ml/min (**Table 1**). Changes in important parameters during the follow-up are detailed in **Table 1**. He has a history of hepatitis B for over 20 years and has been receiving long-term treatment with Entecavir, during which no HBV DNA replication was detected.

**Table 1:** Important parameters changes during follow-up.

Case	Follow-up period (months)	Parameter	Normal range	Base line	2 months	4 months	6 months	9 months	10 months	12 months
Patient 1	7*	Urinary protein (mg/day)	28-141	3156	1895	1333.8	1273	–	–	–
		ALB (g/L)	40-55	42	45	47	46	–	–	–
		Creatinine (mmol/L)	57-97^&^	156	158	–	145	–	–	–
		eGFR(ml/min)	80-120	47.8	47	–	51.9	–	–	–
		IgA	0.82-4.53	3.08	1.92	–	1.8	–	–	–
		urinary red blood cells(numbers/HP)	<3	14	1	1	0	–	–	–
Patient 2	12^#^	Urinary protein (mg/day)	28-141	2016	528	426	392	192	–	80
		ALB (g/L)	40-55	38	46	–	45	47	–	49
		Creatinine (mmol/L)	41-73^&^	53	51	–	51	53	–	53
		eGFR(ml/min)	80-120	143	145	–	145	143	–	143
		IgA	0.82-4.53	6.28	2.7	–	2.6	2.2	–	–
		urinary red blood cells(numbers/HP)	<3	158	28	5	0	0	–	0

“-” No relevant data; ALB, albumin; HP, per high power microscope; eGFR (MDRD), Estimated glomerular filtration rate.

*Telitacicept is used in combination with prednisone and mycophenolate mofetil (MMF).

#The therapy using telitacicept was discontinued after a duration of 9 months.

&The normal range of creatinine is related to age and sex.

Patient 2: A 19-year-old female patient of Chinese ethnicity was diagnosed with anaphylactoid purpura in October 2020. The clinical manifestations included a lower limb rash with no clear trigger, a urine test indicating urinary protein (++), and an erythrocyte count of 10-15 per high power field. She was treated with anti-allergy therapy after refusing a kidney biopsy. Follow-up examinations revealed persistent hematuria and proteinuria. Purpura reappeared and she was admitted to our department on July, 2023. Laboratory examination results were as follows: albumin 47 g/L, creatinine 50 µmol/L, eGFR 145.8 mL/min, PCR 729.6 mg/g, ACR 500 mg/g. Renal pathology findings included diffuse mesangial proliferation, no crescent or interstitial lesions, and segmental sclerosis affecting 3 of the 17 glomeruli. Immunofluorescence showed that IgA was deposited in the mesangial area. Electron density deposition in the mesangial region was observed by immunoelectron microscopy. The renal pathologic diagnosis was “Henoch-Schonlein nephritis IIIb”.

Losartan was given for treatment. During the reexamination on October 26, 2023, UPE was 2016mg/24h, PCR 1865.1mg/g, ACR 1483mg/g, urinary red blood cells 158/HP, albumin 38g/L, creatinine 53umol/L, eGFR 143ml/min, and IgA 6.28 g/L. She declined glucocorticoid therapy, so Telitacicept was administered subcutaneously at 160 mg weekly to treat IgAV nephritis. After two months of treatment with Telitacicept, UPE was 528mg/24h, urinary red blood cells 28/HP, albumin 46g/L, creatinine 51umol/L, eGFR 145ml/min, IgA 2.7g/L (**Table 1**). Upon follow-up at six months, UPE had decreased to 392mg/24h. After 9 months of treatment with Telitacicept, laboratory tests revealed a UPE of 192mg/24h, albumin of 47g/L, creatinine at 53umol/L, eGFR of 143ml/min, and IgA at 2.20g/L. After 9 months, she discontinued the use of Telitacicept, and currently, proteinuria and hematuria are negative, with stable renal function (**Table 1**).

## Discussion

The clinical course of IgAV in adults seems to differ from that in children, particularly because adults face a higher risk of developing end-stage renal disease (ESRD). There is limited data available to guide the best treatment practices for adults with IgAV nephritis. IgAV is a type of systemic vasculitis characterized by the deposition of IgA1 immune complexes in the mesangium. The optimal treatment for IgAV nephritis is currently controversial. While corticosteroid (GC) therapy is commonly used during the acute phase of IgAV, some early studies have shown that controversy has existed as to whether GCs can prevent the development of kidney involvement, reduce its severity or both in IgAV nephritis (8, 9). Patients with IgAV nephritis who exhibit significant kidney disease—such as macroproteinuria, reduced kidney function, crescents, or segmental lesions on kidney biopsy—may be treated with immunosuppressive agents and glucocorticoids (GCs). One study found that combining GCs with cyclophosphamide (CTX) did not offer any benefits over using GCs alone regarding outcomes such as reduced proteinuria, improved renal function, and decreased incidence of ESKD after 12 months (10). An Observational Study illustrated that all 12 patients with sever IgAV nephritis achieved remission of proteinuria and normalization of kidney function at 12 months after accepted MMF and GCs treatment (11). A retrospective study of 53 adult patients with IgAV nephritis found that combining MMF with low-dose prednisone resulted in lower relapse rates compared to high-dose prednisone treatment (12). A single-center retrospective study of 16 patients indicated that Tacrolimus (TAC) combined with GCs may be an effective treatment for IgAV nephritis, particularly among those refractory to CTX or those treated in combination with MMF (13). In this study, 37.5% of patients achieved complete remission (CR), 31.2% achieved partial remission (PR) (13). However, evidence for the treatment of IgAV nephritis is limited and of low quality. Identifying appropriate drug targets is essential for effectively treating IgAV nephritis.

IgAV is an immune-mediated hypersensitivity disease with complex and poorly understood pathophysiological mechanisms. Gd-IgA1 deposits in the vascular walls and mesangium, triggering renal damage by binding to the extracellular transferrin receptor (TfR) on mesangial cells, creating a positive feedback loop (14). Moreover, there is a significant increase in circulating B cells in patients diagnosed with IgAV. Current knowledge suggests that B cells may serve as a therapeutic target to reduce the production of aberrant antibodies against Gd-IgA1. Currently, a few reports analyze the efficacy of Rituximab (RTX: anti-CD20 antibody) in treating IgAV nephritis. A multicenter observational study involving 22 adult patients with refractory or recurrent IgAV nephritis demonstrated that either RTX monotherapy or its combination with additional immunosuppressive therapies led to a 91% remission rate at 6 months (15). Furthermore, it effectively decreased proteinuria by fivefold and stabilized renal function, with only one patient developing ESRD (15). A long-term follow-up (33.7 months) study involving 12 patients showed the efficacy of RTX in aggressive IgAV showed that eleven patients (91.7%) achieved a clinical response at 6-month follow-up and ten patients (83.3%) had a complete response (CR) (16). Two patients needed an additional dose of RTX after 12 months for persistent proteinuria (1g/24h) (16). This study confirms that RTX is both effective and safe for the induction and maintenance of long-lasting IgAV nephritis (16).

The treatment that specifically targets B cells has demonstrated effectiveness in clinical studies. BAFF and APRIL, proteins involved in the development and activation of B lymphocytes, are also promising therapeutic targets. Several novel agents are being investigated for the treatment of IgAN, including those targeting APRIL: Sibeprenlimab, Atacicept, Telitacicept, Povetacicept, and Zigakibart. Among these agents, Sibeprenlimab is a humanized IgG2 monoclonal antibody specifically designed to bind to and neutralize APRIL. In the Phase 2 clinical trial of Sibeprenlimab for IgAN, results indicated that after 12 months of treatment, participants experienced nearly a 50% reduction in 24-hour urinary protein levels, while renal function remained stable (17). BION-1301 is a new humanized monoclonal antibody that inhibits a ligand known to promote cell proliferation (APRIL). A Phase 1/2 study (NCT03945318) is currently underway to evaluate BION-1301. Two dual inhibitors, Atacicept and Telitacicept, are currently being evaluated in Phase II studies for their effectiveness in treating patients with IgAN. Currently, numerous reports highlight the effectiveness of Telitacicept in treating IgAN.

A Phase II clinical trial evaluated the clinical response during the treatment of Telitacicept through detecting changes of IgAN-related serum markers: Gd-IgA1, IgA-containing immune complexes (7). Gd-IgA1 decreased by 43.9% and 50.4% in the 160 mg group and 240 mg group after 24 weeks of treatment, respectively. IgG-IgA immune complex and poly-IgA immune complex showed a dose dependent decreased: the 240mg group had a more than 20% greater decline than the 160mg group. The reduction of proteinuria was consistent with the decrease of IgA immune complex (7). Another Randomized phase 2 trial found that, after 6 months of treatment, mean proteinuria decreased by 49% in the group receiving 240 mg (14 patients) and by 25% in the group receiving 160 mg (16 patients) (4). A retrospective case-control study (6) and a retrospective multicenter study (5) also confirmed that Telitacicept is effective in reducing proteinuria and stabilizing renal function in patients with IgAN. All these studies demonstrated that Telitacicept reduces proteinuria in patients with IgAN, and some studies suggested that it may also increase eGFR. Given the established efficacy and safety of Telitacicept in treating IgAN, along with the similarities between IgAN and IgAV nephritis, we tried to administer Telitacicept to patients with IgAV nephritis, yielding promising results. One patient with initial IgAV nephritis was treated with 160mg Telitacicept and achieved PR after 2 months and CR after 9 months. Another patient with recurrent refractory IgAV nephritis was treated with 160 mg Telitacicept combined with glucocorticoid and MMF and achieved PR after 6 months and stabled kidney function.

Telitacicept is generally well-tolerated, with a few severe adverse reactions(0-10%) reported, and with injection site reactions(6.2-63.3%), irritated skin near the injection site(2.1%), infections(2.1-36.6%), significant decreases in serum immunoglobulin levels(2.1-33.3%), Hyperuricemia(13.3%), and fatigue(5%) in patients with IgAN (4–6). Telitacicept was well tolerated in our patients and no adverse reactions were observed during follow-up.

High quality evidence or guidelines in the treatment of severe IgAV nephritis are lacking. The optimal treatment for IgAV require further clinical investigation. Our study demonstrates that Telitacicept effectively and safely treats adult IgAV nephritis, offering more treatment options for this condition. However, before considering APRIL and BAFF as therapeutic targets, preclinical studies are necessary to understand their roles in IgAV nephritis.

## Data Availability

The original contributions presented in the study are included in the article/supplementary material. Further inquiries can be directed to the corresponding author.
